# Editorial: Embodied bounded rationality

**DOI:** 10.3389/fpsyg.2023.1235087

**Published:** 2023-08-10

**Authors:** Riccardo Viale, Shaun Gallagher, Vittorio Gallese

**Affiliations:** ^1^Department of Economics, University of Milano-Bicocca, Milan, Italy; ^2^University of Memphis, Memphis, TN, United States; ^3^Department of Medicine and Surgery-Unit of Neuroscience, University of Parma, Parma, Italy

**Keywords:** bounded rationality, embodied cognition, enactivism, problem-solving, decision-making

In the last 25 years, a new foundational perspective has emerged in the cognitive sciences under the title of embodied cognition. The core of embodied cognition can be expressed by the general hypothesis that cognitive processes are fundamentally rooted in the morphological traits and sensorimotor and affective systems of the human body. Thinking is based primarily on modal embodied processes rather than amodal ones. These lines of research more or less explicitly recognize the centrality of the embodied variables in economic psychology. This Research Topic aims to demonstrate that the adaptive and ecological dimensions of bounded rationality can be better analyzed by assuming an embodied cognition perspective. Several of the articles in this Research Topic consider how embodied-enactive models of cognition, and the notion of embodied rationality, compare with Herbert Simon's bounded rationality.

Viale et al., in their article “Bounded rationality, enactive problem-solving and the neuroscience of social interaction” aim to show that there is an alternative way to explain human action with respect to the bottlenecks of decision-making psychology. This topic shows that the alternative route recovers the tradition of bounded rationality and problem-solving of Newell and Simon and inserts it into the new research agenda of embodied cognition. According to Simon, the center of gravity of the rationality of the action lies in the ability to adapt. Using the language of embodied cognition, this adaptivity is concerned with the possible solutions implemented to address environmental tasks and problems. From this point of view, the new term, enactive problem-solving, summarizes this fusion between the two moments and could well represent the phenomenon. Problem-solving takes place in a dynamic relationship of an enactive type in a problem space. Within it, repeated feedback allows you to gradually shape the solution. Enactive problem-solving is achieved through the bodily and neural mechanisms typical of embodied cognition, such as the mirror neuron system. Its adaptive function seems effective both in practical and motor tasks and in abstract and symbolic ones. Enactive problem-solving also seems to be able to explain the underlying mechanisms of embodied bounded rationality. Petracca in his article, “Embodying bounded rationality: From embodied bounded rationality to embodied rationality,” considers that embodied rationality is associated with the more radical forms of enactive-embodied cognition, which suggests a genuine transformation in our concept of the rational. He considers the relationship between bounded rationality and the concept of embodied rationality drawn from the multiple views found in embodied cognition literature. He argues that a range of such embodied views, from moderate to radical versions, can inform a new understanding of bounded rationality, which, in Simon's traditional conception, tends to be disembodied. Taking Simon's concept as a “conceptual yardstick,” beginning at zero, Petracca sets out to measure how embodied bounded rationality can get. The concepts of embodied cognition are also fundamental for explaining the mechanisms underlying the adaptive heuristics of rational ecology. This also seems to be confirmed by Gigerenzer in his article “Embodied Heuristics.” He introduces the concept of embodied heuristics, i.e., innate or learned rules of thumb, that exploit evolved sensory and motor skills to facilitate superior decisions. For example, the Gaze Heuristic solves coordination problems from catching prey in flight to catching a frisbee. Several species have adapted this heuristic to their specific sensorimotor abilities, such as vision, echolocation, running, and flying. Exaptation may explain the evolutionary mechanism that led humans to use gaze heuristics to solve tasks beyond their original purpose, for example, in rocket technology. In addition, Mastrogiorgio et al. in their article “More Thumbs than Rules: Is Rationality an Exaptation?” argue that the adaptive mechanisms of evolution are not sufficient for explaining human rationality and positing that human rationality presents exaptive origins, where exaptations are traits evolved for other functions or no function at all, and later co-opted for new uses. They propose an embodied reconceptualization of rationality—embodied rationality—based on the reuse of the perception–action system, where many neural processes involved in the control of the sensory-motor system, salient in ancestral environments, have been later co-opted to create—by tinkering—high-level reasoning processes, employed in civilized niches. They conclude by claiming the non-neutrality of biological endowment for the specification of cognitive processes.

According to Gigerenzer, the deepening of the embodied characteristic of the gaze heuristic is paradigmatic for the study of embodied cognition in relation to ecological rationality. This concept is also reaffirmed by Nordli and Todd in their article “Embodied and embodied ecological rationality: A common vertebrate mechanism for action selection underlies cognition and heuristic decision-making in humans.” They argue that evolution by natural selection has produced an impressive diversity, from fish to birds to elephants, of vertebrate morphology; yet, despite the large species-level differences that otherwise exist in the brains of many animals, the neural circuits that underlie motor control exhibit a functional architecture that is virtually unchanged in every living vertebrate species. The cortico-basal-ganglia-thalamo-cortical (CBGTC) circuitry or loop regulates the embodied pursuit of goals and the learning of embedded goal-pursuit protocols that are custom-molded to fit and exploit structural regularity in the environment. It appears to facilitate motor control, trial-and-error procedural learning, and habit formation. There is evidence to suggest that this same functional circuit has been further adapted to regulate cognitive control in humans and motor control. CBGTC loop may be applied to elimination by aspect and to recognition-based heuristics and can explain the adaptive aspects in the concept of ecological rationality.

The embodied dimension of cognition is developed by Felin and Koenderink, in their article “A generative view of rationality and growing awareness.” They propose the concept of generative rationality as an alternative to bounded and ecological rationality. Generative rationality steers away from conceiving rational agents as “intuitive statisticians” in favor of understanding them as “probing organisms.” They argue that the statistical and cue-based logic of ecological rationality originates in a misapplication of concepts from psychophysics, such as signal detection or just-noticeable differences. They demonstrate this by considering the city-size task. Generative rationality, rather than building on statistics, builds on biology and the concepts of salience and relevance that are characteristic of the pragmatic intentionality (cues-for-something) intrinsic to perception. In addition, this has implications for understanding the emergence of novelty in economic settings. This leads them to offer a modification of Simon's “scissors” metaphor for bounded rationality. This critique of Simon's bounded rationality is also connected with that of Lee in his article “What can deep neural networks teach us about embodied bounded rationality.” He argues that Simon's “bounded rationality” is the principle that humans make decisions based on step-by-step (algorithmic) reasoning using systematic rules of logic to maximize utility. This algorithmic dimension which can be equated to the Turing-Church calculus seems to provide no basis for the interactive and feedback dimension of human cognition, especially at the social level. Instead, the principle of embodied cognition suggests that human decision-makers make use of feedback mechanisms for many of their cognitive functions, including rational decision-making. In this respect, deep neural networks, which have led to a revolution in artificial intelligence, are both interactive and fundamentally non-algorithmic. Their ability to mimic some cognitive abilities much better than previous algorithmic techniques based on symbol manipulation provides empirical evidence of the power of embodied bounded rationality.

A classic way to study social interaction is the experimental use of game theory. In general, the behavioral approach to the study of games is distant from the embodied dimension. Lerique, on the other hand, in his article “Embodied rationality through the glasses of game theory: an empirical touchpoint,” asks the question of how to understand embodied rationality with respect to game theory and bounded rationality. He develops a game-theoretic description of an enactive interaction arrangement (the Perceptual Crossing Paradigm—PCP) to compare with more traditional game-theoretic approaches. In this regard, he considers experimental PCP as a characterization of minimal interaction in which agents coordinate their movements without predetermined instructions. In game theory terms, this is a game of assurance, which is solved *via* the sensorimotor interactions of the agents. This allows game-theoretical approaches to be compared with enactive approaches involving participatory sense-making and embodied interaction. From this point of view, his proposal is linked to that of enactive problem-solving by Viale et al.. The sensorimotor dimension of social interaction is more explanatory than decision-making models based on information and symbolic processing psychology.

Embodied cognition has manifested its explanatory ability not only in practical problem-solving or social interaction but also in abstract thinking and reasoning. Some authors claim that conceptual features of higher-order thinking are grounded on embodied-environmental content. There are many examples: Notions of a set seem to derive from the perception of a collection of objects in a spatial area; recursion builds upon repeated action; derivatives (in calculus) make use of concepts of motion, boundary, etc. Some authors provide a number of examples of advances in mathematics inspired by bodily and socially embedded practices: counting leading to arithmetic and number theory; measuring to calculus; shaping to geometry; architectural formation to symmetry; estimating to probability; moving to mechanics and dynamics; and grouping to set theory and combinatorics. The article of Michirev et al. “A Developmental Embodied Choice Perspective Explains the Development of Numerical Choices” is on the same wavelength. It addresses the topic of the embodiment of decision-making from a developmental perspective, where the body provides cues used in abstract choices. In particular, they consider choices in numerical settings in which the body is not necessarily needed for the solution, like the magnitude-judgment task. They propose a developmental trajectory for developmental turning points at which fingers and hands become cues. Cue validity increases through frequent and successful use over the course of development. The authors conclude that when the base-10 system is introduced, it builds upon our sensorimotor system and its cues.

Embodied cognition theories are generally opposed to dualistic models of the mind. The 4E cognition approach, i.e., embodied, enactive, extended, and embedded cognition, is holistic about the mental dimension. The embodied dimension of bounded rationality excludes the possibility of a separation between Type 1 and Type 2 processes of the mind. On the contrary, Bellini-Leite argues in his article “Dual Process Theory: Embodied and Predictive; Symbolic and Classical” that dual process theory is currently a popular theory for explaining why we show bounded rationality in reasoning and decision-making tasks. According to him, a problem for this theory is identifying a common principle that ties the features T1 and T2 together, explaining how they coordinate to express a common output. Taken together, various reasons have been given to hold this hypothesis in relation to representational format, automaticity, working memory, and speed. Psychological research must verify whether the hypotheses that the T1 responses derive from predictive processing and the T2 responses follow a classical analytic architecture are valid. Experiments with artificial intelligence can test whether this hybrid is useful and feasible. Neuroscience should be able to detect what kind of mechanisms are interrelated in classical reasoning, judgment, and decision-making tasks. Bellini-Leite hypothesis that connects to embodied cognition is that it seems likely that these mechanisms will be found not so much in the brain region but most likely in the action potentials of motor activity.

Another consideration of an epistemic type is proposed by Arfini and Magnani, in their article “Embodied irrationality? Knowledge avoidance, willful ignorance and the paradox of autonomy.” They argue that knowledge avoidance and willful ignorance, although often treated as identical, should be distinguished as falling into different categories of the epistemic spectrum. They adopt an epistemic and embodied perspective to clarify the difference between these concepts. Specifically, they define willful ignorance as an irrational pattern of reasoning and, in contrast, knowledge avoidance as epistemically rational in some circumstances. They consider a variety of phenomena, such as wishful thinking, self-deception, and akrasia, and the impact of epistemic feelings, to show how knowledge avoidance can be considered a rational, autonomy-increasing strategy.

How does embodied cognition and its explanatory role in decision-making fit into the ontological representation of reality? Mousavi and Sunder in their article “Emergence and Embodiment in Economic Modeling” introduce a three-tier structure with physics at the bottom, biology at the center, and socio-psychology at the top level. Their structure characterizes the familiar modeling method of economics by specifying social-psychological preferences and goals to construct an objective function, specifying the opportunity set by constraints, and then seeking the optimal choice of action from the set. It is represented as an approach that originates in the outer part of reality with the possibility of proceeding to the biological center, which uses the principles of the physical core to derive its formalization. The three-level structure organizes principles from the physical, biological, and social sciences, proposing a new, broader, non-reductionist perspective on human behavior. The objectives of embodied cognition correspond to a method of investigation from the center to the outside.

## Author contributions

All authors listed have made a substantial, direct, and intellectual contribution to the work, and approved it for publication.

